# Immunotherapy – 2066. A study on skin prick test patterns of atopic patients managed in a tertiary care setting of Sri Lanka

**DOI:** 10.1186/1939-4551-6-S1-P149

**Published:** 2013-04-23

**Authors:** Anoma Siribaddana, Fariha Sitheeque, Sonali Surangi, Srinath Illeperuma, Dinesh Dassanayaka

**Affiliations:** 1 Respiratory Medicine, Teaching Hospital Kandy, Kandy, Sri Lanka; 2 Teaching Hospital Kandy, Sri Lanka; 3 Department of Physiology, Faculty of Medicine, Peradeniya, Sri Lanka

## Background

Studies of common allergens causing allergic rhinitis and other atopic conditions in Sri Lanka are scarce. We used skin prick testing to study the pattern of allergic sensitization in Sri Lankan patients having allergic rhinitis.

## Methods

We performed skin prick test (SPT) using 19 allergens on 54 patients having allergic rhinitis (AR). AR was classified according to AREA classification^1^. Presence of coexisting Asthma and allergic conjunctivitis was documented. The severity of asthma was assessed using the GINA guidelines. Informed written consent was obtained by a member of the research team. SPT was performed using a panel of 19 allergens. Positive control was done using a standard solution of histamine and the negative was performed with glycerol buffer. The resultant induration was measured using a standard scale and recorded in millimeters. A wheel of 3 mm was considered as positive. The results were analyzed in percentages.

## Results

We studied 25 males and 29 females. The age range of patients was from 09 to 68 yrs with a mean age of 33.5yrs. A positive reaction to at least one allergen was seen in 87.1% of patients. Intermittent Rhinitis was seen in 53.7% and persistent disease was seen in 46.3%. Moderate to severe disease was found in72.2% and the rest had mild disease. Coexisting asthma was seen in 43.6% and conjunctivitis was seen in 29.6% of the study population. The results of SPT expressed in percentages showed, Dermatophagoides farina spp. 59.3%, Dermatophagoides pteronyssinus spp. 63%, Wheat Dust 33%, Cotton Gin 33%, Rice Dust 14%, Aspergillus flavus 18.5%, Aspergillus fumigates 7.4% Aspergillus niger 11.1%, Aspergillus glaucus/amstelodami 11%, Tricho. mentagrophytes 7.4%, Candida spp. 18.5%, Cladosporum herbarium 11%, Epidermo floccosum 22.2%, German Cockroach 51.9%, American Cockroach 40.7%, Bermuda Grass 11%, Red Mulberry 7.4%, White Mulberry 11.1% and Acacia 3.7% positivity.

## Conclusions

The commonest allergen sensitizing this cohort of patients was of the Dermatophagoides pteronyssinus, followed by the Dermatophagoides farina. Next common were the German Cockroach 51.9% and American Cockroach 40.7%. The least common was of Acacia.

